# Selection of Multi-Drug Targets against Drug-Resistant *Mycobacterium tuberculosis* XDR1219 Using the Hyperbolic Mapping of the Protein Interaction Network

**DOI:** 10.3390/ijms241814050

**Published:** 2023-09-13

**Authors:** Noor ul Ain Zahra, Aimilia-Christina Vagiona, Reaz Uddin, Miguel A. Andrade-Navarro

**Affiliations:** 1 Lab 103 PCMD ext., Dr. Panjwani Center for Molecular Medicine and Drug Research, International Center for Chemical and Biological Sciences, University of Karachi, Karachi 75270, Pakistan; noozahra@uni-mainz.de; 2 Institute of Organismic and Molecular Evolution, Faculty of Biology, Johannes Gutenberg University, Hans-Dieter-Hüsch-Weg 15, 55128 Mainz, Germany; avagiona@uni-mainz.de

**Keywords:** drug resistance, drug targets, protein–protein interaction network, network hyperbolic mapping

## Abstract

Tuberculosis remains the leading cause of death from a single pathogen. On the other hand, antimicrobial resistance (AMR) makes it increasingly difficult to deal with this disease. We present the hyperbolic embedding of the *Mycobacterium tuberculosis* protein interaction network (mtbPIN) of resistant strain (MTB XDR1219) to determine the biological relevance of its latent geometry. In this hypermap, proteins with similar interacting partners occupy close positions. An analysis of the hypermap of available drug targets (DTs) and their direct and intermediate interactors was used to identify potentially useful drug combinations and drug targets. We identify *rpsA* and *rpsL* as close DTs targeted by different drugs (pyrazinamide and aminoglycosides, respectively) and propose that the combination of these drugs could have a synergistic effect. We also used the hypermap to explain the effects of drugs that affect multiple DTs, for example, forcing the bacteria to deal with multiple stresses like ethambutol, which affects the synthesis of both arabinogalactan and lipoarabinomannan. Our strategy uncovers novel potential DTs, such as *dprE1* and *dnaK* proteins, which interact with two close DT pairs: arabinosyltransferases (*embC* and *embB*), Ser/Thr protein kinase (*pknB*) and RNA polymerase (*rpoB*), respectively. Our approach provides mechanistic explanations for existing drugs and suggests new DTs. This strategy can also be applied to the study of other resistant strains.

## 1. Introduction

Tuberculosis (TB) is a chronic and deadly infectious disease and one of the top ten leading causes of death worldwide. TB occurs in every country of the world and affects all age groups. It is also a leading cause of death in HIV-positive patients [1]. The “Global Tuberculosis Report” from the World Health Organization (WHO) reported 1.45 million deaths and ten million individuals infected with TB in 2019. An increasingly alarming cause of concern is latent tuberculosis infection caused by MDR strains of *Mycobacterium tuberculosis* (MTB). An estimated 0.5 million cases of multi-drug-resistant TB (MDR-TB) were reported in 2019, of which 186,772 were fully diagnosed and only 57% received treatment [2]. To make matters worse, the current unprecedented COVID-19 pandemic has produced major direct and indirect negative impacts on TB control programs [3,4]. The major impact of the pandemic on tuberculosis was a decline in the diagnosis and reporting of TB cases. According to the WHO 2021 report on TB, there was a decline of 18.3% in newly diagnosed TB cases, i.e., a reduction from 7.1 million reported cases in 2019 to 5.8 million cases in 2020 [4]. In 2020, TB alone caused 1.5 million deaths, comparable to the 1.8 million deaths from COVID-19 in the same year [5].

To deal with the threat of MDR-TB, the scientific community studies several topics: the development of bacterial resistance [6], understanding the biology of MTB [7], discovering and validating new TB DTs, the identification of inhibitors with new mechanisms of action, formulating adequate anti-TB regimens, improving the standard therapy duration, and developing better and faster diagnostics and innovative biological assays as in vivo microenvironment representations [5]. The current regimen to treat TB uses various drugs and fixed-dose combinations: (i) the two-drug regimen: isoniazid and rifampicin; (ii) the three-drug regimen: isoniazid, rifampicin, and pyrazinamide; and (iii) the four-drug regimen: ethambutol, pyrazinamide, isoniazid, and rifampicin. These combinations are intended to reduce the emergence of drug resistance. MDR-TB is treated with fluoroquinolones, clofazimine, linezolid, amoxicillin, and clavulanic acid combinations. Bedaquiline, pretomanid, and delamanid are three new drugs that have been approved for clinical use within the last 50 years [1].

Despite these efforts, TB infection still poses serious threats due to a rapid increase in antimicrobial resistance (AMR). This requires new antibiotics to which bacteria are not yet resistant [8]. Over the past few years, conscious and concerted efforts have been made to find new pairs of targets and inhibitors. However, since 1984, no new class of antibiotic (with a new mechanism of action) has been introduced [9], which means that there is a void of 30 years in anti-TB drug development. As a result, the pool of drugs available against TB is rapidly dwindling with the increase in AMR [10].

The systematic analysis of cellular networks offers possibilities to obtain insights into biological function. Since proteins are interconnected in a network of interactions to perform specific cellular functions, disease mechanisms might be related to subsets of interacting proteins [11]. Protein interactions often mediate diseases or bacterial infections [12,13]. The study of the underlying molecular mechanisms of disease in terms of protein interaction networks can help us better understand disease progression, etiology, pathogenesis, and resistance mechanisms, and aid in identifying druggable targets [14]. Protein–protein interaction network mapping approaches have been used to reveal mechanisms of disease, indicating new therapeutic options [15].

Recent applications of network theory to protein interaction networks are opening new avenues for the exploration of the mechanisms of disease. Network theory is a tool for modeling diverse types of complex systems (cellular interactions, internet, social, and economic networks) [16]. There are several algorithms and models that support the fact that the topology of complex networks, such as the human protein–protein interaction network (hPIN), is shaped by an underlying hidden geometry [17]. One of the widely accepted models is the popularity–similarity model (PSM), representing the two-dimensional hyperbolic space (H^2^) in a disk [18]. In the PSM, the network evolves by the continuous appearance of new nodes in the hyperbolic plane with logarithmically increasing radial coordinates and uniformly random angular coordinates. A new node establishes connections to the previous ones with a probability depending on the hyperbolic distance. The tendency of a node to connect to hyperbolically close nodes arises from a trade-off between its popularity (radial coordinate) and its similarity (angular coordinate) compared to the newly arriving node [19]. In parallel, another field closely related to hyperbolic embedding has recently received a great boost with the development of hyperbolic networks [20,21,22]. Alanis-Lobato et al. performed the embedding of the hPIN to hyperbolic space and found biological interpretations of the embedded network in terms of the PSM. They concluded that the radial position of the nodes captures information about the conservation and age of proteins. In contrast, their angular position reflects the functional and spatial organization of the proteins in the cell [17]. This mapping may help to understand the complexity of different disorders. Recently, mapping hPIN to H^2^ led to a better understanding of a complex human disorder such as Huntington’s disease [23].

This line of research motivated us to apply hyperbolic embedding to the interaction network of bacterial proteins to understand its biological relevance. We followed an interolog approach to construct the MTB protein interaction network (mtbPIN) of a resistant strain (MTB XDR1219), followed by embedding interactions in the two-dimensional hyperbolic plane to explore, analyze, and comprehend the network.

We focused on the protein–protein interactions of the available DTs from a large mtbPIN embedded in the H^2^. We hypothesized that detecting two or more DTs hyperbolically close to each other in H^2^ could be used to search for one, two, or more different drugs, simultaneously inhibiting them and providing us with an understanding of the molecular mechanism of the effect of those drugs from a geometric perspective. We also studied the role of common interactors of DT pairs geometrically lying close in the H^2^ involved in the same or different pathways to understand the complexity of resistance and tolerance mechanisms of available drugs.

## 2. Results

### 2.1. Network Embedding on the Hyperbolic Disc

First, we generated a protein–protein interaction network from the high confidence interaction data [24]. The network embedding is only possible with the network’s largest connected component (LCC). The LCC of the mtbPIN comprised 20,487 interactions between 2880 proteins, or nodes mapped out of 3883 proteins of the pathogen’s proteome. This network was embedded by applying the LaBNE + HM algorithm in the hyperbolic space H^2^ [17,25,26] (see Section 5 for details). Afterward, the hyperbolic coordinates of each node were inferred to analyze the geometrical properties of the network.

### 2.2. Protein Clustering in the Angular Similarity

**Figure 1:**
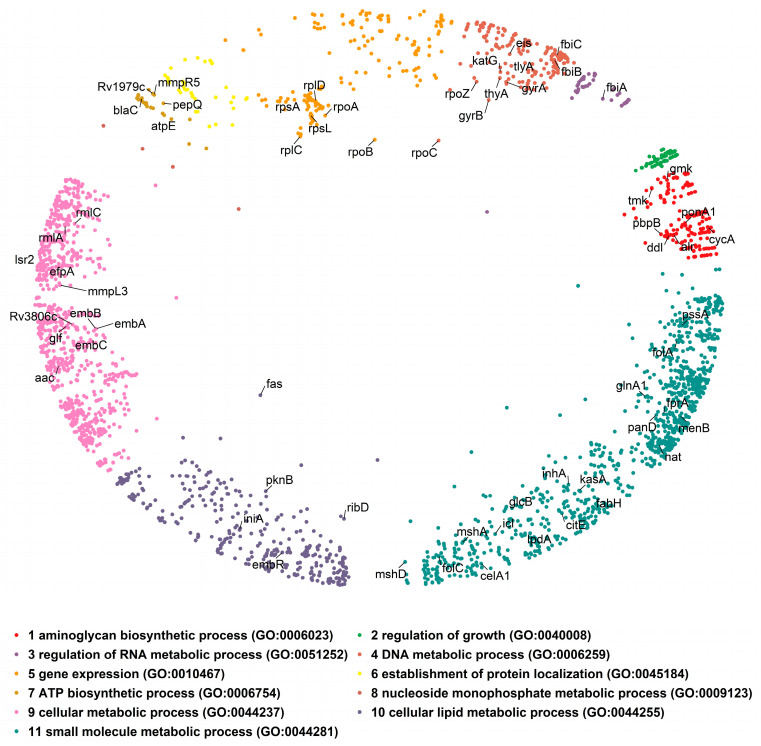
The embedded mtbPIN in a two-dimensional hyperbolic disc. Different colors represent protein clusters identified in the angular dimension of the hyperbolic space by large gaps separating them. Individual clusters were analyzed for enrichment in GO terms (biological process) to reveal their biological relevance. The positions of DTs (nodes labelled with gene symbol) in the hyperbolic map are indicated; DTs were found in most clusters.

One of the two interesting attributes of the PSM is the similarity component, which corresponds to the angular coordinate of the nodes in the hyperbolic plane and reflects characteristics that make a node similar to others [17]. To exploit the biological meaning of the angular dimension, we find proteins grouped in clusters by identifying gaps between consecutive inferred angles (θ; Supplementary Figure S1A, see Section 5 for details). This resulted in eleven protein clusters in the mtbPIN. The identified protein clusters revealed the cell’s functional organization, which is supported by performing the Gene Ontology (GO) term enrichment of the proteins in individual clusters. The proteins are clustered in a similarity-based fashion as each cluster is found to be enriched with various aspects of the GO biological process (Figure 1). We focused on understanding the interactions of proteins that are validated DTs. The 63 DTs were found distributed in nine of the eleven clusters of the MTB hyperbolic network (Figure 1). Only cluster 2 “regulation of growth” and cluster 8 “nucleoside monophosphate metabolic process” lacked any DTs. The coordinates of this map are available in Supplementary Table S1.

### 2.3. Hyperbolic Distance and Drug–Target Interactions

Once the biological meaning of the similarity component of the PSM had been interpreted, we used both components (*r* and θ) to compute the hyperbolic distance (dH^2^) between the 63 MTB DTs and their adjacent proteins in the hyperbolic map. According to the coordinates inferred by LaBNE + HM, if two proteins are close to each other, the probability that they will interact is high, while if the proteins are far away, the chances of interaction are lower [17]. Accordingly, we observed that proteins separated by a shorter dH^2^ made plausible interactions, and fewer direct interactions were observed with the greater dH^2^ values (Supplementary Figure S1B). The shortest distance between 63 DTs and each protein in the network was also computed to determine intermediate interactors between DTs. We analyzed direct interactors of DTs with other DTs that can be in the same or different regions of the mtbPIN. Secondly, we studied the cases in which DT pairs share a common neighbor/interactor(s) that are not yet exploited as DTs but could be involved in the same pathways as DTs. We propose that these could be novel, potentially effective DT candidates.

#### Drug Targets and Their Interactors in H^2^

**Figure 2:**
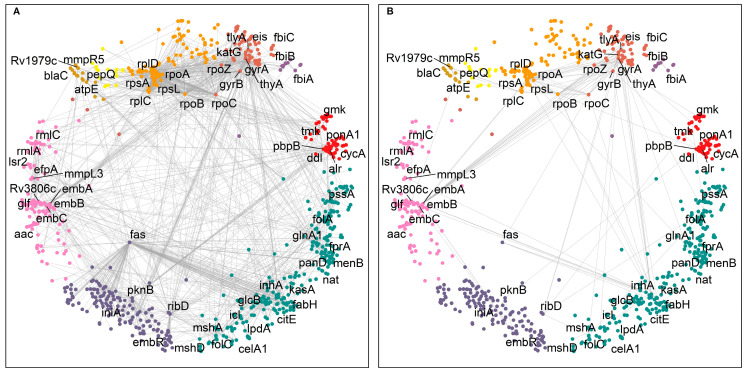
Interaction network of 63 DTs in hyperbolic space. (**A**) The small cumulative network of DTs making 1808 interactions. (**B**) A total of 153 direct interactions between DTs. DT interactors are found located in the same and in different clusters. The DTs are positioned in nine of the eleven clusters and labelled with their gene symbol—cluster colors as in Figure 1.

We focused our analysis on the small cumulative network of all DTs and their direct interactors. The DTs were observed to be involved in 1808 interactions (Figure 2A), including, to our surprise, 153 interactions between them (Figure 2B). For example, the MTB RNA polymerase (RNAP) responsible for transcription consists of multiple subunits α_2_ββ’ω encoded by *rpoA*, *rpoB*, *rpoC*, and *rpoZ*, respectively. These subunits interact to make the molecular assembly of RNAP and are reported as DTs targeted by rifampicin and its derivatives (Figure 2) [27]. Figure 2 shows the DTs positioned in nine of the eleven clusters and their direct interactors in ten of the eleven clusters. Cluster 2 “regulation of growth” (Figure 1, small green cluster at the top right) has no DT or direct interactor, strengthening the idea that this function remains an unexplored area for drug targeting.

To understand the information contained in the latent geometry of the mtbPIN about the mode of action of DTs and the possible role of their common interactors in bacterial resistance and drug tolerance, we analyzed the DTs that are hyperbolically close by considering the following cases: (i) DTs that are hyperbolically close and are involved in same, related, or unrelated metabolic pathways or processes; (ii) DTs that share a common interactor (the shortest path length between DTs is d_s_ = 2); and (iii) DTs that are geometrically close but connected by a longer shortest path (d_s_ > 2).

### 2.4. Hyperbolically Close DTs

We looked for hyperbolically close DTs, irrespective of their interaction profile. Supplementary Table S2 represents the list of DT pairs in order of increasing hyperbolic distance. We found that DT pairs that are hyperbolically close were also making direct interactions (d_s_ = 1). In addition, the location of DT pairs in the relevant clusters was determined. A total of 309 pairs of DTs were found to be located in the same protein clusters, and 64 pairs were direct interactors, while 1644 DT pairs were found in different protein clusters, and among them, 89 DT pairs were direct interactors (Supplementary Figure S2). We observed that DTs making interactions with other DTs are highly interconnected and scattered throughout the hyperbolic network (Figure 2B), except for the cluster 2 “regulation of growth” (compare to Figure 1).

**Figure 3:**
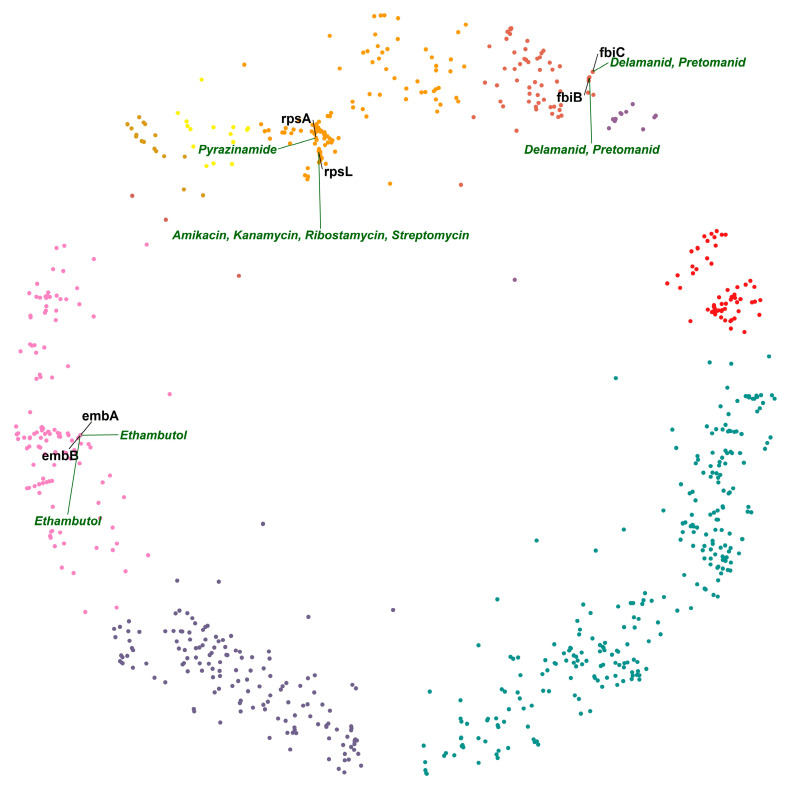
Hyperbolically close DT pairs present in their relevant clusters. The members of each of the two DT pairs, *embA–embB* and *fbiB–fbiC*, are targeted by the same drugs (ethambutol and delamanid/pretomanid, respectively), whereas the members of the DT pair *rpsA–rpsL* are inhibited by different drugs: pyrazinamide and aminoglycosides—cluster colors as in Figure 1.

Next, we studied pairs of interacting DTs using the hyperbolic map and its properties to locate pairs in proximity or within relevant clusters. In the hyperbolic map of MTB, we found that DTs *embB* and *embA* are hyperbolically the closest. *embA* and *embB* are co-transcribed and function together as a heterodimer [28,29]. Emb proteins are proposed to be the target of the anti-tuberculosis drug ethambutol, which inhibits arabinosyltransferases and, consequently, cell wall biogenesis [28]. The second DT pair on the list is *fbiC* and *fbiB* (Figure 3), which are associated with activating the prodrugs delamanid and pretomanid. Both drugs are second-line TB drugs (SLTDs). They participate in inhibiting the biosynthesis of methoxy and keto mycolic acid through the F_420_ mycobacterial system and nitrous oxide generation. Mutations in *fbiA*, *fbiB*, and *fbiC* genes have been linked to altered production of F_420_ and resistance to drugs targeting them [30]. The third DT pair on the list is interesting because two different drugs target it. *rpsA* and *rpsL* are hyperbolically close and in the same cluster (cluster 5 “gene expression”; Figure 3). *rpsA* is a molecular target of pyrazinamide [31], and *rpsL* is a molecular target of aminoglycosides [32]. *rpsL* and *rpsA* are ribosomal proteins involved in protein biosynthesis. The two different classes of drugs acting on them produce their action by inhibiting protein synthesis. We hypothesize that a combination of pyrazinamide and one of the aminoglycosides could have a synergistic effect, given that they would target DTs closely related in the mtbPIN.

**Figure 4:**
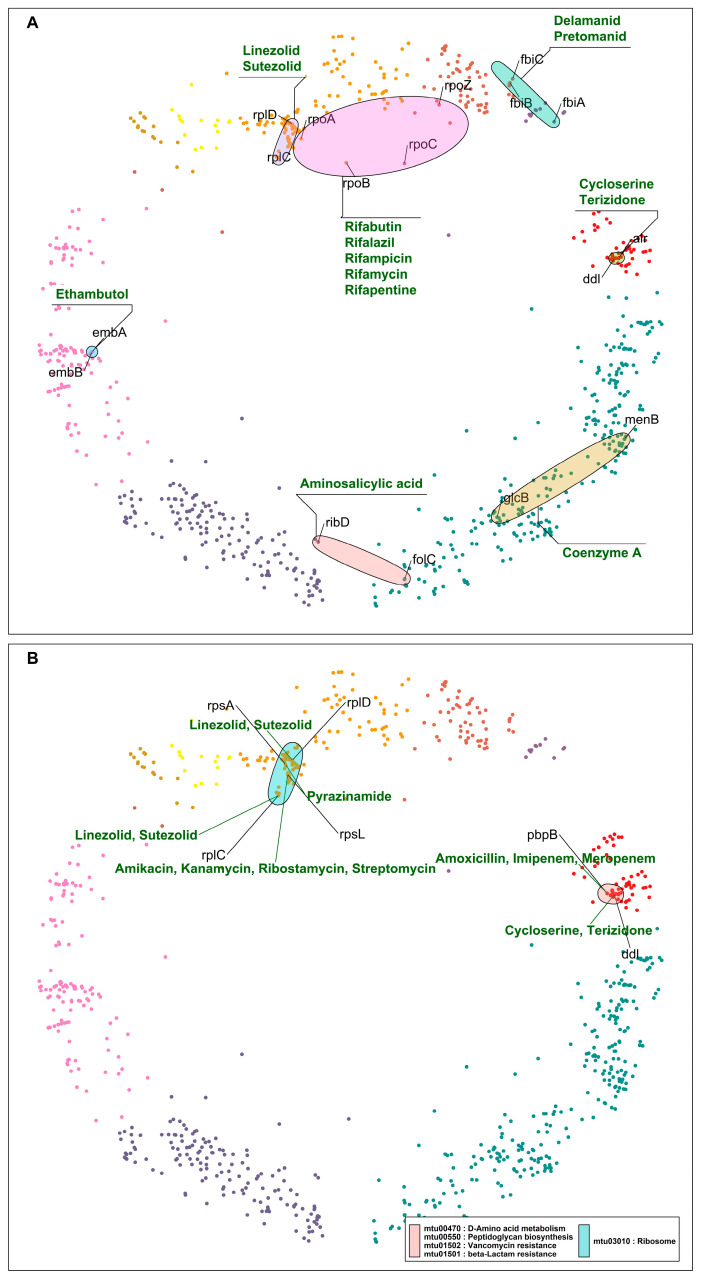
(**A**) DT pairs hyperbolically close, inhibited by the same class of drugs, and involved in the same pathways, processes, or complexes. The colored ellipses represent DT pairs or groups targeted by the same drugs, for example, ribosomal proteins participating in protein synthesis inhibited by linezolid and sutezolid; similarly, DT pairs part of the RNA polymerase complex targeted by rifampicin and its derivatives. (**B**) DT pairs that are components of the same pathways and are inhibited by two different classes of drugs. Drug classes and gene symbols are indicated—cluster colors as in Figure 1.

To find potentially effective drugs and drug combinations acting on DT pairs, we searched for hyperbolically close DT pairs (i) targeted by the same drug (Table 1; Figure 4A) or (ii) involved in the same pathway but targeted by different drugs (Table 2; Figure 4B).

Close DT pairs targeted by the same class of drugs (Table 1; Figure 4A) represent drugs whose effects are potent because they affect many related genes that are probably involved in related protein interactions (according to their short distances in the mtbPIN). These DTs are functionally related proteins that are targeted by antibiotic compounds that bind to two or more distinct DTs, e.g., DNA-directed RNA polymerase proteins are inhibited by rifampicin and its derivatives. Similarly, *ddl* (alanine ligase) and *alr* (alanine racemase) are targeted by cycloserine, which affects the peptidoglycan biosynthesis in the MTB cell wall (Figure 4A).

Close DT pairs involved in one pathway but targeted by different drugs highlight the groups of drugs that could have a synergistic effect by acting in multiple proteins of the same pathway (Table 2; Figure 4B). The first five hyperbolically close DT pairs are ribosomal proteins targeted by aminoglycosides, pyrazinamide, and repurposed anti-TB drugs linezolid and sutezolid [33]. The sixth DT pair in the list is *pbpB* and *ddl*. *pbpB* inhibitors are amoxicillin, imipenem, and meropenem, whereas *ddl* is inhibited by cycloserine and terizidone (Figure 4B). Both proteins are part of the peptidoglycan biosynthesis pathway, a bacterial cell wall component. These anti-TB drugs target different proteins in the same pathway, possibly producing a synergistic effect [34].

**Figure 5:**
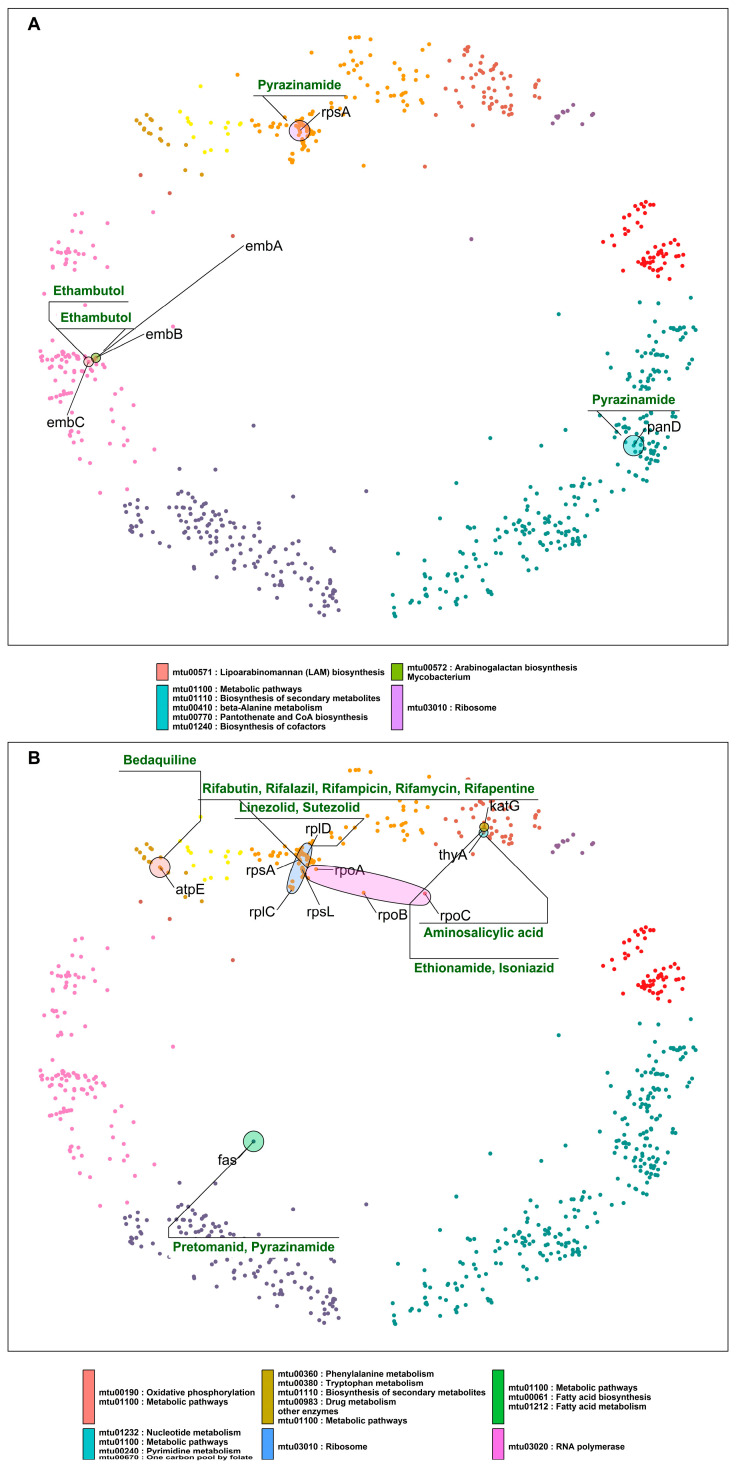
(**A**) DT pairs that are components of different pathways and are inhibited by the same drugs. (**B**) DT pairs that are components of different pathways and are inhibited by different drugs. Ellipses indicate pathways and group nodes, labelled with gene symbols representing DT pairs and annotated with drugs acting on them—cluster colors as in Figure 1.

Next, we highlight DT pairs that are not closely related components or part of the same molecular pathway or process but are nevertheless inhibited by the same drug (Table 3; Figure 5A). In such cases, the bacteria must deal with two different stress responses, hampering the bacterial ability for efficient antibiotic stress adaptability, providing these antibiotics with an additional advantage. Ethambutol inhibits proteins (*embABC*) involved in arabinogalactan and lipoarabinomannan (LAM) biosyntheses. Other cases in this category are *rpsA* and *panD*, which are direct interactors located in two clusters (4 “DNA metabolic process” and 11 “small molecule metabolic process”, respectively), comparatively hyperbolically distant (Table 3 and Figure 5A). However, the proposed mechanism of action of pyrazinamide by binding to *rpsA* and inhibiting the trans-translation process has been directly questioned or contradicted [35], whereas it inhibits bacterial coenzyme A biosynthesis by binding to aspartate decarboxylase (*panD*) [36].

The last category of DT pairs we considered are also not closely related components of the same pathways and are targeted by different drugs (Table 4; Figure 5B). Ribosomal proteins are mainly found hyperbolically close and making interactions with RNA polymerase proteins *rpoA* and *rpoB* (Table 4). The first pair is *rpoA* and *rplD*. *rpoA* is the DNA-dependent RNA polymerase subunit alpha, involved in the transcription of DNA to RNA, and it is inhibited by rifampicin and its derivatives. *rplD* is the 50S ribosomal protein L4 participating in the translation of proteins; the drugs linezolid and sutezolid prevent the assembly of the functional 70S initiation complex by binding to the 23S ribosomal RNA of the 50S subunit. This inhibits protein production, preventing the bacteria from multiplying [37].

In the case of *rpoA* and *rplD*, the corresponding drugs inhibit DTs located in the same cluster but involved in unrelated processes. This results in a drug synergistic effect. However, in the mtbPIN map, we can find DT pairs with very distant proteins such as *fas* (fatty acid synthase) and RNA polymerase proteins (*rpoC*, *rpoB*), which are located in two different clusters and are a part of two different pathways. *fas* are a component of fatty acid biosynthesis and metabolism pathways, which pretomanid and pyrazinamide inhibit. The inhibition of *fas* by pyrazinamide has been contradicted [38], as in the case of *rpsA*. But rifampicin and pyrazinamide are co-prescribed to treat resistant TB along with isoniazid and ethambutol. The combination has shown to be effective in treatment outcomes, which could result from a drug synergistic effect. On the other hand, the target specificity of pretomanid is still elusive but inhibits mycolic acid synthesis [39]. We found other DT pairs in the map with distant proteins *rplC*-*fas* and *rplC*-*atpE*, targeted by linezolid/pretomanid and linezolid/bedaquiline, respectively. These groups of drugs inhibit components of protein synthesis (linezolid) [40], ATP biosynthesis (bedaquiline) [41], and mycobacterium cell wall biosynthesis (pretomanid) [39]. Figure 5B elaborates these cases, where synergistic drug action is observed between drugs that inhibit unrelated pathways or processes.

#### Common Interactors between DTs

**Figure 6:**
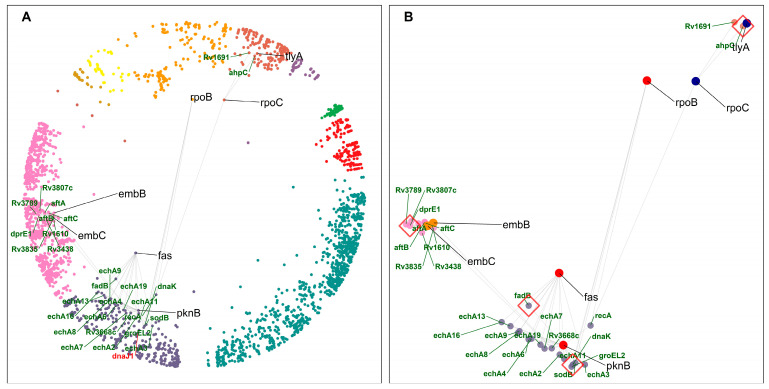
Common interactors of DTs. (**A**) DT pairs (black gene labels) with their respective common interactors (green gene labels) in the hyperbolic disc. *dnaJ1* (labelled with red font), found hyperbolically close to *dnaK*, has been highlighted for discussion (see text for details)—cluster colors as in Figure 1. (**B**) DT pairs and common interactors. Red diamonds highlight common interactors discussed in the text. *dnaK* and *ahpC* impart resistance to their interacting DTs, whereas *dprE1* and *fadB* are involved in mycolic acid biosynthesis and fatty acid degradation, respectively.

To study common interactors of DT pairs, we collected information related to DT pairs that have d_s_ = 2 and share an interaction with a protein that is not a DT. Anti-TB drugs target different essential pathways, processes, and complexes in bacteria, but the current drug resistance and tolerance have made TB treatment challenging. Therefore, we looked for pairs of DTs that were hyperbolically close on the map and shared a common interactor hinting at additional candidates for drug targeting that could work synergistically with already existing DTs. Figure 6A,B show an overview of common interactors in the mtbPIN hyperbolic map.

The closest DT pair is fatty acid synthase (*fas*) and Ser/Thr protein kinase (*pknB*), sharing functionally similar MTB enoyl coA hydratase (Ech homologs) proteins and fatty acid degradation (*fadB*) protein as common interactors (Supplementary Table S3). FAS/polyketide synthase biosynthetic pathways are responsible for mycolic acid synthesis, a unique main constituent of the mycobacterial cell wall. The MTB *ech* proteins and *fadB* are components of the fatty acid degradation process. This DT pair and its common interactors are components of related pathways, fatty acid metabolism and degradation, in cluster 10 “cellular lipid metabolic process”.

Another DT pair, *embC* and *embB*, share similar proteins as common interactors, which are involved in lipoarabinomannan (LAM) biosynthesis, such as *dprE1* (Decaprenylphosphoryl-beta-D-ribose oxidase). *embC–dprE1–embB* are participating in the related pathway, and all shared common interactors of this DT pair are located in cluster 9 “cellular metabolic process”. Our results suggest that other members of this pathway, such as *dprE1*, could be potentially efficient DTs.

*tlyA* (16S/23S rRNA (cytidine-2′-O)-methyltransferase) is one of the targets of capreomycin and shares *ahpC* (Alkyl hydroperoxide reductase C) as a common interactor with the DT *rpoC* (DNA-directed RNA polymerase subunit beta). *ahpC* overexpression imparts resistance to isoniazid [42]. This protein protects MTB against oxidative stress during in vitro and in vivo infections and is a virulence factor. It also enables bacteria to grow within the host macrophages (by resisting ROS/RON) and regulates the host immune responses [43].

We found that *dnaK* is a common interactor of the DT pair RNA polymerase (*rpoB*) and *pknB*. DnaK is a chaperone involved in protein folding and maintaining protein integrity with the help of its cochaperones (DnaJ1 and DnaJ2) in MTB [44]. *dnaK* and *dnaJ1* are hyperbolically close and found in the same cluster of the mtbPIN map (Figure 6A). *dnaK* is part of the RNA degradation pathway, while *rpoB* is associated with the RNA polymerase pathway targeted by Rifampicin, a first-line TB drug (FLTD). Recently, a study showed that *dnaK* associates with MTB DTs and proteins in metabolic pathways targeted by antimicrobial agents [44]. They were found to directly stabilize the stress imparted by resistance-conferring amino acid substitutions in DTs. *dnaK* was found to be associated with mutant alleles in MTB with clinically relevant amino acid substitutions in the *rpoB* gene. MTB chaperones can be studied further as potential DTs by inhibiting the *dnaK* system to prevent or sensitize drug resistance in MTB [44].

### 2.5. DTs Geometrically Close with d_s_ > 2

**Figure 7:**
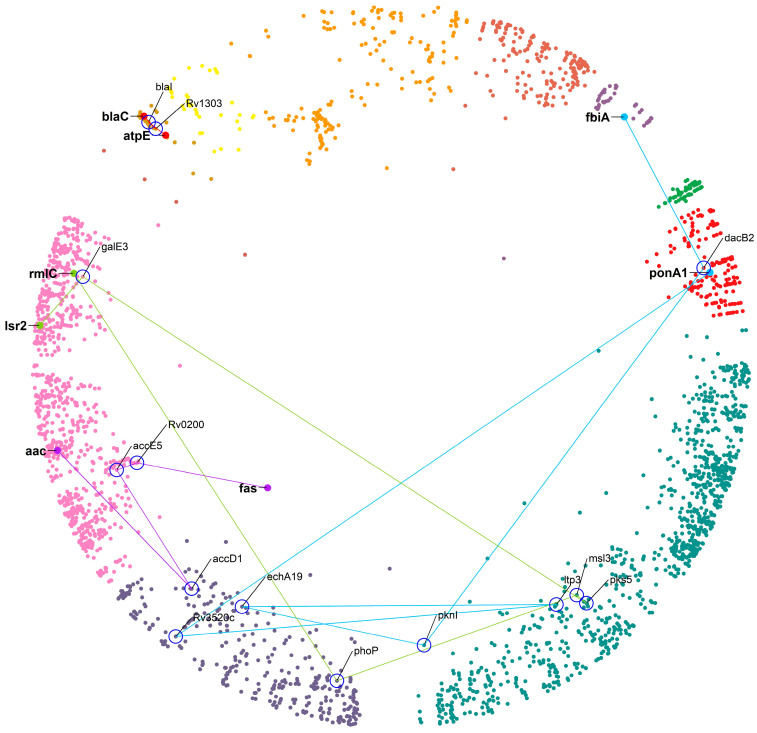
The four shortlisted DT pairs hyperbolically close and connected by the shortest paths d_s_ = 3 to 6. The DT nodes are labelled with a gene symbol (bold) and highlighted with a large size. Each path and the nodes connecting the DT pairs are represented by different colors with nodes labelled with gene symbols (not bold) and surrounded by a blue circle—cluster colors as in Figure 1.

Finally, we checked for DT pairs lying hyperbolically close but with the shortest paths between DT pairs of higher length (d_s_ > 2). We only studied four cases selected from each group of d_s_ (d_s_ = 3, d_s_ = 4, d_s_ = 5, d_s_ = 6) with the smallest value of hyperbolic distance. Table 5 and Figure 7 elaborate on these cases. Other cases can be easily found in the Supplementary Tables.

The closest DT pair with d_s_ = 3, *atpE-blaC*, is linked by two proteins: *blaI* and *Rv1303*. *blaC* is targeted by beta-lactamase inhibitors (amoxicillin + clav), resulting in the inhibition of peptidoglycan biosynthesis and *atpE*, which is the F_o_ unit of the ATP synthase, targeted by bedaquiline [45]. Amoxicillin (a beta-lactam antibiotic) shows anti-mycobacterial activity in combination with beta-lactamase inhibitors (clavulanic acid), referred to as augmentin. From the literature, we found that the *blaI*, along with *whiB4* (a protein involved in detecting when cells are under stress and that regulates the expression levels of mycothiol and enzymes responsible for breaking down the antibiotic drugs), are responsible for providing underlying tolerance to beta-lactamase inhibitors [46]. This example illustrates that even a two-step connection between DTs could result in interesting novel DT suggestions. A factor that could be a positive influence, in this case, is that the hyperbolic distance between the DT pair is low, as well as the distances between the proteins in the connecting path, and all the proteins involved remain within the same cluster (see Figure 7 for an overview). The longer the paths, the more we expect the hyperbolic distances to be greater with positions in different clusters and lower the chances that the proteins in the connecting path will be relevant DTs. The following examples illustrate this for the best possible case regarding the distance between the DTs for a given connection path length.

For a path of d_s_ = 4, the closest DT pair is *aac–fas* linked by *accD1*/*accE5*/*Rv0200* proteins. Aminoglycoside 2’-*N*-acetyltransferase (*aac*) modifies aminoglycosides through co-enzyme A-dependent acetylation of the 2- hydroxyl or amino group, conferring resistance to the DT pair [47]. The linking proteins *accD1* and *accE5* are acetyl-CoA carboxylases (ACC) that catalyze the α-carboxylation of acetyl-CoA to produce malonyl-CoA, which serves as a building block for fatty acid biosynthesis [48], whereas *Rv0200* is a possible transmembrane protein. All these proteins remain in a relatively similar region of the hyperbolic map, which could mean that a protein of unknown function, such as *Rv0200*, could be a good DT.

For a path of d_s_ = 5, the closest DT pair is *Isr2–rmlC* linked by *galE3*/*pks5*/*msl3*/*phoP* proteins. Lsr2 is a nucleoid-associated protein (NAP) of MTB and RmlC is dTDP-4-dehydrorhamnose 3,5-epimerase, both identified as high confidence DTs. For a path of d_s_ = 6, the closest DT pair is *fbiA–ponA1* linked by *dacB2*/*pknI*/*echA19*/*ltp3*/*Rv3520c*. This DT pair is inhibited by pretomanid and amoxicillin, respectively. We found no evidence in the literature that these two drugs are in use as a combination. In these two last cases, while the DT pair belongs to a similar region of the hyperbolic map, the steps connecting them involve proteins of the large clusters 10 “cellular lipid metabolic process” and 11 “small molecule metabolic process”. We suggest that these connections are not very specific and that their importance for the regulation of cellular processes in which the DTs are involved will not be very relevant, not supporting the intermediate proteins as DTs.

## 3. Discussion

The two-dimensional hyperbolic embedding of different networks is both useful and relevant. The hyperbolic embedding of biological networks, such as human protein interaction networks, has been studied to uncover their biologically relevant latent hyperbolic geometry. Our previous study showed that the two components of the PSM, i.e., the radial coordinates of the proteins (nodes), correlate with their evolutionary age, with proteins that evolved earlier (older) in the evolutionary process tending to be closer to the center of the hyperbolic plane, while younger proteins lie on the plane’s periphery. Proteins with related biological functions and cellular localizations cluster along the angular coordinates [17]. These distributions have useful meaning in terms of protein networks as the older proteins tend to have a more significant number of interactions and are shifted closer to the center of the map, while proteins with similar functions tend to be part of the same pathways and complexes, meaning that they are more interconnected, and tend to cluster in the same region of the map.

In this work, we performed the two-dimensional hyperbolic embedding of a bacterial protein interaction network. The embedding of the mtbPIN behaved similarly to the hPIN, allowing us to study the mapping of MTB (drug-resistant XDR1219 strain) protein–protein interactions with biological relevance. We focused on the map properties of proteins that are validated DTs. The 63 DTs were found distributed in nine clusters of the MTB hyperbolic network (Figure 1). The underlying hyperbolic geometry of DT pairs and their common interactors were used to study their functionality in their mode of action and antibiotic tolerance and resistance, respectively. We considered the cases where DT pairs are (i) hyperbolically close, (ii) located in the same or different clusters, (iii) part of the same or related or different metabolic pathway or processes, and (iv) targeted by the same or different drugs. We found that the current anti-TB agents produced their action by inhibiting proteins involved in metabolic processes or pathways vital for bacterial survival. The antimycobacterial drugs rifampicin, ethambutol, delamanid, pretomanid, cycloserine, and terizidone produce their action by inhibiting DT pairs of functionally related proteins of the same molecular processes or metabolic pathways. Rifampicin and ethambutol are part of the first line of TB drug regimen to manage the infection.

TB is managed with drug combinations; we found that two different classes of drugs target DT pairs involved in more than one metabolic pathway. Drug combinations are always designed to have a synergistic effect. Cycloserine inhibits the D-alanine pathway for peptidoglycan biosynthesis, increasing susceptibility to beta-lactam inhibitors [49]. However, drug antagonism is also reported when linezolid is given in combination with pyrazinamide and isoniazid due to their harmful interaction that reduces absorption [50]. We found that drugs like ethambutol and pyrazinamide target DT pairs that are part of different metabolic pathways, producing an efficient antibiotic effect [34]. On the other hand, DT pairs in different pathways are targeted by two different drug classes, resulting in synergistic effects. Pretomanid is given in combination with linezolid and bedaquiline to treat XDR-TB, and this combination has demonstrated a high treatment success rate [51].

Despite decades of intensified research to understand TB and its cure, the disease continues to burden the world population. TB treatment requires the administration of multiple antibiotics for a longer duration (several months). Approved anti-TB drugs treat drug sensitive (DS), MDR, and XDR TB. Drug resistance phenotypes and drug-tolerant bacterial populations complicate treatment outcomes. Therefore, we searched for common interactors of DT pairs that could be studied as alternative and new stronger DTs with robust modes of action. Mycolic acid biosynthesis involves numerous key enzymes such as the FAS system, fatty-acid-modifying enzymes, fatty-acid-activating and condensing enzymes, along with transporters and transferases that are potential drug targets [1]. In the case of resistant MTB strains causing MDR-TB and XDR-TB, mycolic acid biosynthesis pathways offer a great source of alternative potential drug targets for developing antimicrobial drugs [1]. The regulation of the MTB cell wall biogenesis needs to be better understood. Recently, Ser/Thr protein kinases (STPKs) have appeared in the molecular picture of cell wall biogenesis as a major regulatory mechanism [52]. The phosphorylation mediated by STPKs has been reported to inhibit many enzymes involved in the synthesis of mycolic acids [53]. *pknB* is among the major regulators of STPK-mediated signaling in the MTB [52]. *dprE1* is identified as a common interactor of the DT pair *embC–embB*, involved in lipoarabinomannan (LAM) biosynthesis. LAMs are glycolipids in the bacterial cell wall that contribute to virulence, support bacterial survival, and prevent host defense mechanisms in several ways (phagosome maturation, immune cell activation, antigen presentation, and regulation of cytokines). DprE1 has been extensively exploited as a potential new stronger DT in the field of anti-TB drug development [1]. Expression levels of many bacterial genes increase in response to drug-induced stress. Capreomycin targets the mechanism of protein synthesis by inhibiting ribosomal proteins. A study performed by Miryala et al. [42] showed that exposure to capreomycin upregulated a set of genes, including *ahpC*, which codes for an alkyl hydroperoxide reductase that implicates resistance to reactive nitrogen and oxygen intermediates, making it easier for the bacteria to develop resistance against isoniazid [54]. It is reported that the overexpression of genes like *ahpC*, *kasA*, *ndh*, and efflux pumps such as *mmpl7*, *mmpl3*, *efpA*, and *mmr*, along with mutations in *katG*, contribute resistance toward FLTDs. Eighty-five percent of rifampicin resistance strains are also resistant to isoniazid [55]. Rifampicin resistance is mainly due to mutations in the *rpoB* gene [56]. These *rpoB*, *katG*, and *ahpC* genes are interrelated. Hence, targeting any of their products in the early stages of infection could increase the extent of infection control [57].

Antimicrobial agents often target and inhibit enzymes that catalyze essential cellular processes, leading to selection pressure that results in the emergence of mutants with resistance conferring amino acid substitutions in targets. These mutations provide a selective advantage to bacteria in the presence of drugs, but can also lead to protein instability, changes in enzyme activity, or result in the development of additional stress, compromising bacterial fitness [58]. Bacteria with antimicrobial resistance mutations might be fit enough to divide, grow, and survive through compensatory mutations, but other general mechanisms may also alleviate the fitness cost of mutations [59,60]. Fay et al. found that protein chaperones *dnaK* (hsp70 homolog) with its cochaperones *dnaJ1* and *dnaJ2* support the mutations in *rpoB* by directly stabilizing the mutant RNA polymerase [44]. *dnaK* is a common interactor of different DTs (*gyrA*, *gyrB*, *rpoB*, and *pknB*) in the mtbPIN, but DNA topoisomerases are not found to be its direct clients. This could mean that either mutations in them do not alter the bacterial physiology enough to require chaperone buffering or that other chaperone systems could be responsible for buffering the mutational effects. Accordingly, in the mtbPIN, *gyrB*, *rpoB*, *kasA*, *katG*, and *pknB* share *groEL2* (homolog of Hsp60) as a common interactor (Supplementary Table S3). Experimental studies are desired to validate these points. Many studies have reported the role of protein chaperones in supporting amino acid substitutions in diverse organisms and cell types [61,62,63,64,65]. Hence, protein chaperone systems can be targeted for inhibition to sensitize or prevent drug resistance in MTB.

Furthermore, by exploiting the hyperbolically closest DT pairs with d_s_ > 2, we found the role of *blaI* in drug tolerance. When MTB is exposed to augmentin, cell wall damage occurs, perturbing membrane integrity and thereby affecting various cellular processes, such as the respiratory chain, ATP generation, and redox balance. These imbalances in cellular processes result in metabolic instability and effective drug-induced killing. To tolerate augmentin, the bacteria shift their respiration to an energetically poor route involving NADH dehydrogenase 2 (NDH2) and cytochrome BD oxidase (CyBD). This redirection causes the generation of ROS (reactive oxygen species), which is neutralized by intramycobacterial redox buffer (mycothiol) to protect MTB from augmentin. This oxidative shift in the MTB is a signal to calibrate the expression levels of beta-lactamases, peptidoglycan synthesis enzymes, antioxidants, carbon metabolism, and alternative respiration via WhiB4. A study by Mishra et al. found that oxidized WhiB4 binds and represses *blaC* and *blaR*, whereas reduction reversed this effect [46]. The *blaI* (transcriptional regulator BlaI) binds to the promoter of *blaC* and of the genes encoding CyBD and ATP synthase [66], and their expression levels were high in the absence of WhiB4. They found that a loss of WhiB4 derepressed *blaR* and stimulated as well as expressed *blaC*, possibly through the proteolytic cleavage of BlaI by BlaR (having protease activity). Their findings indicated that the presence of WhiB4 made the bacterial cell more susceptible to augmentin therapy by reducing the production of enzymes that break down beta-lactam drugs and mycothiol. They suggested that the cross-talks between *whiB4* and *blaI* pathways result in antibiotic tolerance. It is suggested that augmentin could be more effective against MDR and XDR tuberculosis if combined with drugs that can change the levels of ROS (reactive oxygen species) inside the MTB cells [46]. We found a DT geometrically close to *blaC* in the hyperbolic map, *atpE*, which is targeted by bedaquiline, one of the antibiotics that disturbs ATP homeostasis. Hence, bedaquiline could be an effective combination to produce a synergistic effect by potentiating the action of beta-lactams and beta-lactamases in MTB.

With our work, we have illustrated how to use the hyperbolic mapping of a pathogen’s PIN to study the synergistic effects of drugs. Intriguingly, several of the proteins we identified are already being investigated for their drug therapy potential [67,68,69,70], which endorses the usefulness of interpreting the mtbPIN in searching for biologically meaningful interactions and interactors of DTs.

## 4. Materials and Methods

### 4.1. Dataset Construction

The proteome of MTB-resistant strain XDR1219 was retrieved from the FTP site of the NCBI RefSeq [71]. The model resistant strain MTB H37Rv protein–protein interactions were obtained from the STRING v11.5 database (https://string-db.org/ (accessed on 1 July 2023)) [72].

Available DTs of the current therapeutic anti-TB regimen of MTB and information on their respective drugs were retrieved from the DrugBank Database v. 5.1.8 (https://go.drugbank.com/ (accessed on 1 July 2023)) [73] and Therapeutic Target Database (http://db.idrblab.net/ttd/ (accessed on 1 July 2023)) [74].

### 4.2. Prediction of the MTB Protein–Protein Interaction Network (mtbPIN)

The PPI network was generated based on information obtained from the model organism MTB H37Rv. The interactions were predicted based on high throughput experimental evidence (>0) and combined score (≥0.7) obtained from the STRING database v 11.5. STRING aims to harbor all known and predicted associations between proteins. These associations include both physical and functional associations [72]. The interaction data were processed to remove redundancy. The proteome of MTB XRD1219 was retrieved from the NCBI RefSeq server (ftp://ftp.ncbi.nlm.nih.gov/ (accessed on 1 July 2023)). Interologs were defined based on sequence similarity to proteins of the model strain MTB H37Rv (using BLASTp) to predict the interactions of this resistant strain of MTB. Interologs are the conserved interaction between a pair of proteins whose orthologs interact in another species. Orthology relations were used to derive interologs in MTB XDR1219 to interacting proteins in MTB H37Rv [75]. The resulting network consisted of 2916 nodes (out of 3883 MTB XDR1219 proteins) and 20,500 interactions, excluding self-loops and duplicate edges (interactions).

### 4.3. Mapping of the mtbPIN in Hyperbolic Space

The mtbPIN was embedded in two-dimensional hyperbolic space (H^2^). The network embedding was performed with the R package “NetHypGeom” [76]. It implements the LaBNE + HM algorithm, an approach that combines maximum likelihood estimation [26] and manifold learning [76] to decipher the underlying hyperbolic geometry of networks. The popularity–similarity model (PSM) interprets the hidden geometrical meaning of the network in hyperbolic space. All nodes lie in the hyperbolic disc with polar coordinates (*r* and θ), where hyperbolic distance represents the popularity dimension (*r*) and angular distance, the similarity (θ). The popularity dimension of radial coordinates *r* of nodes determines that the nodes that joined the system first tend to be closer to the center of the hyperbolic plane [18,26]. The largest component of the resultant mtbPIN consisted of 2880 proteins and 20,487 protein–protein interactions between them. The hyperbolic coordinates (*r* and θ) of each protein in the network were inferred with parameters γ = 2.763, T = 0.519, and w = 2π.

### 4.4. Protein Clustering in the Angular Similarity Dimension

The protein clusters in the angular similarity dimension were identified by large gaps separating the protein groups. The nodes were arranged by their increasingly angular coordinate θ, and the difference between θ_i_ and θ_i+1_ was computed to identify the largest gaps between protein clusters in the similarity dimension. Gap size g (g = 0.03398) was chosen to separate the proteins in 16 clusters (Supplementary Figure S1A). Two clusters (clusters 12 and 15) with only one protein and 5 clusters (clusters 1, 2, 5, 12 and 15) that had no Gene Ontology (GO) enrichment (see below) were merged with the next cluster clockwise. This resulted in 11 clusters with GO enrichment.

### 4.5. Gene Ontology Functional Enrichment Analysis

Gene Ontology (GO) enrichment analysis [77] of each protein cluster was performed with the Gene Ontology resource (http://geneontology.org/ (accessed on 1 July 2023)). Only top biological process (BP) terms with a significance level (*p*-value < 0.05) were kept.

### 4.6. Computation of Hyperbolic Distances

The hyperbolic coordinates (*r* and θ) were used to compute the hyperbolic distances (dH^2^) between the DTs and each protein in the hyperbolic map. Similarly, the shortest path distance (ds) between the DTs and each protein in the network was computed. DTs were sorted by their increasing hyperbolic distance (Supplementary Table S2).

## 5. Conclusions

The embedding of mtbPIN in two-dimensional hyperbolic space (H^2^) resulted in a better understanding of mapped protein interactions, leading to results of biological relevance. We focused on the DT pairs hyperbolically close, located in the same or different clusters, involved in the same or different metabolic pathways or molecular processes, and targeted by the same or different drugs. We also investigated the role of common interactors of hyperbolically close DT pairs. We found that proteins targeted by anti-microbial drugs make 153 direct interactions between them, suggesting the mode of action of TB drug combinations, as they target the protein complexes involved in pathways (cell wall biosynthesis, translation, transcription, replication, etc.) essential to bacterial survival. We also found that some common interactors of hyperbolically close DT pairs imparted resistance and provided tolerance to available drugs through different mechanisms that can be considered as potential drug candidates. Our results provide datasets that could be used to find mechanisms of existing and novel DTs, illustrating a procedure that could be applied to other bacteria and resistant strains using the interolog approach. We propose our approach as a contribution to developing new sets of combined drugs to make bacterial resistance increasingly difficult.

## Figures and Tables

**Table 1:** List of DT pairs arranged in increasing hyperbolic distance involved in the same metabolic pathways and inhibited by the same class of drugs.

DT_a DT_b	dH_2_	Pathway of DT_a	Pathway of DT_b	Common Drug
*embB*/*embA*	4.95	mtu00572: Arabinogalactan biosynthesis—Mycobacterium	mtu00572: Arabinogalactan biosynthesis—Mycobacterium	Ethambutol
*fbiC*/*fbiB*	9.49	mtu01100: Metabolic pathways, mtu00680: Methane metabolism, mtu01120: Microbial metabolism in diverse environments	mtu01100: Metabolic pathways, mtu01120: Microbial metabolism in diverse environments, mtu00680: Methane metabolism	Delamanid, Pretomanid
*ddl*/*alr*	12.38	mtu00470: D-Amino acid metabolism, mtu00550: Peptidoglycan biosynthesis, mtu01502: Vancomycin resistance, mtu01100: Metabolic pathways	mtu01502: Vancomycin resistance, mtu01100: Metabolic pathways, mtu00470: D-Amino acid metabolism	Cycloserine, Terizidone
*rpoC*/*rpoB*	14.21	mtu03020: RNA polymerase	mtu03020: RNA polymerase	Rifabutin, Rifalazil, Rifampicin, Rifamycin, Rifapentine
*rpoZ*/*rpoC*	14.63	mtu03020: RNA polymerase	mtu03020: RNA polymerase	Rifabutin, Rifalazil, Rifampicin, Rifamycin, Rifapentine
*rpoB*/*rpoA*	14.65	mtu03020: RNA polymerase	mtu03020: RNA polymerase	Rifabutin, Rifalazil, Rifampicin, Rifamycin, Rifapentine
*rplD*/*rplC*	15.30	mtu03010: Ribosome	mtu03010: Ribosome	Linezolid, Sutezolid
*rpoC*/*rpoA*	16.85	mtu03020: RNA polymerase	mtu03020: RNA polymerase	Rifabutin, Rifalazil, Rifampicin, Rifamycin, Rifapentine
*rpoZ*/*rpoB*	18.16	mtu03020: RNA polymerase	mtu03020: RNA polymerase	Rifabutin, Rifalazil, Rifampicin, Rifamycin, Rifapentine
*rpoZ*/*rpoA*	20.68	mtu03020: RNA polymerase	mtu03020: RNA polymerase	Rifabutin, Rifalazil, Rifampicin, Rifamycin, Rifapentine
*folC*/*ribD*	23.12	mtu00790: Folate biosynthesis, mtu01240: Biosynthesis of cofactors, mtu01100: Metabolic pathways	mtu01240: Biosynthesis of cofactors, mtu02024: Quorum sensing, mtu00740: Riboflavin metabolism, mtu01100: Metabolic pathways, mtu01110: Biosynthesis of secondary metabolites	Aminosalicylic acid
*glcB*/*menB*	25.86	mtu01120: Microbial metabolism in diverse environments, mtu01200: Carbon metabolism, mtu00630: Glyoxylate and dicarboxylate metabolism, mtu01110: Biosynthesis of secondary metabolites, mtu01100: Metabolic pathways, mtu00620: Pyruvate metabolism	mtu01110: Biosynthesis of secondary metabolites, mtu00130: Ubiquinone and other terpenoid-quinone biosynthesis, mtu01240: Biosynthesis of cofactors, mtu01100: Metabolic pathways	Coenzyme A
*fbiB*/*fbiA*	25.96	mtu01100: Metabolic pathways, mtu01120: Microbial metabolism in diverse environments, mtu00680: Methane metabolism	mtu01240: Biosynthesis of cofactors, mtu01120: Microbial metabolism in diverse environments, mtu01100: Metabolic pathways, mtu00680: Methane metabolism	Delamanid, Pretomanid
*fbiC*/*fbiA*	26.21	mtu01100: Metabolic pathways, mtu00680: Methane metabolism, mtu01120: Microbial metabolism in diverse environments	mtu01240: Biosynthesis of cofactors, mtu01120: Microbial metabolism in diverse environments, mtu01100: Metabolic pathways, mtu00680: Methane metabolism	Delamanid, Pretomanid

**Table 2:** List of DT pairs involved in the same pathways inhibited by two different classes of drugs. The DT pairs are arranged in increasing order of hyperbolic distances.

DT_a DT_b	dH_2_	Common Pathway	Common GO Term	Drug_a	Drug_b
*rpsA*/*rpsL*	9.89	mtu03010: Ribosome	5 gene expression (GO:0010467)	Pyrazinamide	Amikacin, Kanamycin, Ribostamycin, Streptomycin
*rpsL*/*rplC*	13.80	mtu03010: Ribosome	5 gene expression (GO:0010467)	Amikacin, Kanamycin, Ribostamycin, Streptomycin	Linezolid, Sutezolid
*rpsL*/*rplD*	14.04	mtu03010: Ribosome	5 gene expression (GO:0010467)	Amikacin, Kanamycin, Ribostamycin, Streptomycin	Linezolid, Sutezolid
*rpsA*/*rplD*	14.32	mtu03010: Ribosome	5 gene expression (GO:0010467)	Pyrazinamide	Linezolid, Sutezolid
*rpsA*/*rplC*	14.49	mtu03010: Ribosome	5 gene expression (GO:0010467)	Pyrazinamide	Linezolid, Sutezolid
*pbpB*/*ddl*	17.04	mtu01501: beta-Lactam resistance, mtu00550: Peptidoglycan biosynthesis	1 aminoglycan biosynthetic process (GO:0006023)	Amoxicillin, Imipenem, Meropenem	Cycloserine, Terizidone

**Table 3:** List of DT pairs at long hyperbolic distances, involved in different pathways and inhibited by the same class of drugs. The DT pairs are arranged in increasing order of hyperbolic distances.

DT_a DT_b	dH_2_	Pathway of DT_a	Pathway of DT_b	Common Drug
*embC*/*embA*	16.62	mtu00571: Lipoarabinomannan (LAM) biosynthesis	mtu00572: Arabinogalactan biosynthesis—Mycobacterium	Ethambutol
*embC*/*embB*	16.63	mtu00571: Lipoarabinomannan (LAM) biosynthesis	mtu00572: Arabinogalactan biosynthesis—Mycobacterium	Ethambutol
*rpsA*/*panD*	25.32	mtu03010: Ribosome	mtu01100: Metabolic pathways, mtu01110: Biosynthesis of secondary metabolites, mtu00410: beta-Alanine metabolism, mtu00770: Pantothenate and CoA biosynthesis, mtu01240: Biosynthesis of cofactors	Pyrazinamide
*thyA*/*ribD*	25.60	mtu01232: Nucleotide metabolism, mtu01100: Metabolic pathways, mtu00240: Pyrimidine metabolism, mtu00670: One carbon pool by folate	mtu01240: Biosynthesis of cofactors, mtu02024: Quorum sensing, mtu00740: Riboflavin metabolism, mtu01100: Metabolic pathways, mtu01110: Biosynthesis of secondary metabolites	Aminosalicylic acid
*folC*/*thyA*	27.73	mtu00790: Folate biosynthesis, mtu01240: Biosynthesis of cofactors, mtu01100: Metabolic pathways	mtu01232: Nucleotide metabolism, mtu01100: Metabolic pathways, mtu00240: Pyrimidine metabolism, mtu00670: One carbon pool by folate	Aminosalicylic acid
*gmk*/*citE*	28.53	mtu01100: Metabolic pathways, mtu01232: Nucleotide metabolism, mtu00230: Purine metabolism	mtu02020: Two-component system	Formic acid
*rmlA*/*tmk*	28.96	mtu00541: O-Antigen nucleotide sugar biosynthesis, mtu00521: Streptomycin biosynthesis, mtu00523: Polyketide sugar unit biosynthesis, mtu00525: Acarbose and validamycin biosynthesis, mtu01250: Biosynthesis of nucleotide sugars, mtu01110: Biosynthesis of secondary metabolites, mtu01100: Metabolic pathways	mtu01100: Metabolic pathways, mtu00240: Pyrimidine metabolism, mtu01232: Nucleotide metabolism	Thymidine

**Table 4:** List of DT pairs involved in different pathways and inhibited by different drugs. The DT pairs are arranged in increasing order of hyperbolic distances.

DT_a DT_b	dH_2_	Pathway of DT_a	Pathway of DT_b	Drug_a	Drug_b
*rpoA*/*rplD*	12.47	mtu03020: RNA polymerase	mtu03010: Ribosome	Rifabutin, Rifalazil, Rifampicin, Rifamycin, Rifapentine	Linezolid, Sutezolid
*rpoA*/*rpsL*	13.97	mtu03020: RNA polymerase	mtu03010: Ribosome	Rifabutin, Rifalazil, Rifampicin, Rifamycin, Rifapentine	Amikacin, Kanamycin, Ribostamycin, Streptomycin
*rpoA*/*rpsA*	14.34	mtu03020: RNA polymerase	mtu03010: Ribosome	Rifabutin, Rifalazil, Rifampicin, Rifamycin, Rifapentine	Pyrazinamide
*rpoA*/*rplC*	14.72	mtu03020: RNA polymerase	mtu03010: Ribosome	Rifabutin, Rifalazil, Rifampicin, Rifamycin, Rifapentine	Linezolid, Sutezolid
*rpoB*/*rplC*	14.93	mtu03020: RNA polymerase	mtu03010: Ribosome	Rifabutin, Rifalazil, Rifampicin, Rifamycin, Rifapentine	Linezolid, Sutezolid
*rpoB*/*rpsL*	15.35	mtu03020: RNA polymerase	mtu03010: Ribosome	Rifabutin, Rifalazil, Rifampicin, Rifamycin, Rifapentine	Amikacin, Kanamycin, Ribostamycin, Streptomycin
*rpoB*/*rplD*	15.73	mtu03020: RNA polymerase	mtu03010: Ribosome	Rifabutin, Rifalazil, Rifampicin, Rifamycin, Rifapentine	Linezolid, Sutezolid
*rpoB*/*rpsA*	15.87	mtu03020: RNA polymerase	mtu03010: Ribosome	Rifabutin, Rifalazil, Rifampicin, Rifamycin, Rifapentine	Pyrazinamide
*thyA*/*katG*	15.94	mtu01232: Nucleotide metabolism, mtu01100: Metabolic pathways, mtu00240: Pyrimidine metabolism, mtu00670: One carbon pool by folate	mtu00360: Phenylalanine metabolism, mtu00380: Tryptophan metabolism, mtu01110: Biosynthesis of secondary metabolites, mtu00983: Drug metabolism—other enzymes, mtu01100: Metabolic pathways	Aminosalicylic acid	Ethionamide, Isoniazid
*fas*/*rpoB*	16.23	mtu01100: Metabolic pathways, mtu00061: Fatty acid biosynthesis, mtu01212: Fatty acid metabolism	mtu03020: RNA polymerase	Pretomanid, Pyrazinamide	Rifabutin, Rifalazil, Rifampicin, Rifamycin, Rifapentine
*rpoC*/*rplC*	16.57	mtu03020: RNA polymerase	mtu03010: Ribosome	Rifabutin, Rifalazil, Rifampicin, Rifamycin, Rifapentine	Linezolid, Sutezolid

**Table 5:** List of four shortlisted DT pairs selected from each group of shortest path length (d_s_) with the smallest hyperbolic distances. NA = not available.

DT_a DT_b	dH_2_	d_s_	Pathway of DT_a	Pathway of DT_b	Drug_a	Drug_b
*atpE*/*blaC*	18.19	3	mtu01110: Biosynthesis of secondary metabolites, mtu00311: Penicillin and cephalosporin biosynthesis, mtu01501: beta-Lactam resistance	mtu00190: Oxidative phosphorylation, mtu01100: Metabolic pathways	Bedaquiline	Amoxicillin
*aac*/*fas*	20.29	4	mtu01100: Metabolic pathways, mtu00061: Fatty acid biosynthesis, mtu01212: Fatty acid metabolism	NA	Coenzyme A, Ribostamycin	Pretomanid, Pyrazinamide
*lsr2*/*rmlC*	25.44	5	mtu00521: Streptomycin biosynthesis, mtu00523: Polyketide sugar unit biosynthesis, mtu00541: O-Antigen nucleotide sugar biosynthesis, mtu01100: Metabolic pathways, mtu01250: Biosynthesis of nucleotide sugars, mtu01110: Biosynthesis of secondary metabolites	NA	Pretomanid	S,S-(2-Hydroxyethyl) Thiocysteine
*fbiA*/*ponA1*	27.19	6	NA	mtu01240: Biosynthesis of cofactors, mtu01120: Microbial metabolism in diverse environments, mtu01100: Metabolic pathways, mtu00680: Methane metabolism	Delamanid, Pretomanid	Amoxicillin

## Data Availability

Not applicable.

## Supplementary Materials

The supporting information can be downloaded at: https://www.mdpi.com/article/10.3390/ijms241814050/s1.
