# Supplementary material for: Selection of Multi-Drug Targets against Drug-Resistant Mycobacterium tuberculosis XDR1219 Using the Hyperbolic Mapping of the Protein Interaction Network
Source: Int J Mol Sci. 2023 Sep 13;24(18):14050. doi: 10.3390/ijms241814050 (PMC10530867; doi:10.3390/ijms241814050)
Supplement: Supplementary file 1 [file ijms-24-14050-s001.zip › SUPPLEMENTARY/Supplementary Legends.pdf]

Supplementary Materials

Title: Selection of multi-drug targets against drug-resistant *Mycobacterium tuberculosis* XDR1219 using the hyperbolic mapping of the protein interaction network

Authors: Noor ul Ain Zahra, Aimilia-Christina Vagiona, Reaz Uddin, Miguel A. Andrade-Navarro

Figure S1. Embedding of the mtbPIN in the hyperbolic space. (A). Identification of big gaps between angular coordinates in the mtbPIN. Proteins were sorted by their angular coordinates (θ) increasingly and the difference between θi and θi+1 was estimated. The peaks in the plot correspond to the gap sizes in the angular dimension and hint at the presence of similarity-based clusters. To determine the start and the end of each cluster, the gap size g = 0.03398, was chosen, resulting in 16 clusters. Clusters with only one protein or without GO enrichment were merged with the consecutive one (clockwise) resulting in 11 clusters. This allowed us to perform meaningful functional enrichment analysis of each group of proteins. (B). The quality of the hyperbolic embedding of the mtbPIN was evaluated by the connection probability against the hyperbolic distance. The fit of the values resulting from the embedding via the LaBNE+HM algorithm to the theoretical values inferred from the Popularity-Similarity Model, indicates that the embedding was of good quality.

Figure S2. The number of DT pairs in the same and different clusters. The number of DT pairs in the same and different clusters. The DT pairs with shortest pathlength ds = 1 are direct interactors, out of which 64 are in the same clusters and 89 are found in different clusters

Supplementary Table S1. The coordinates of the nodes indicating their cluster, and whether they are DTs or direct interactors of DTs. Columns indicate protein identifier (UniprotKb_id, gene symbol), coordinates (r, theta), cluster number, GO Term of clusters, status of protein as DT(Drug target) and NDT (Nondrug target), and direct interactor of DT.

Supplementary Table S2. The list of DT pairs arranged in increasing order of their respective hyperbolic distances. Columns indicate DT pairs (gene symbol), Hyperbolic distance, shortest path length, metabolic pathways, GO Term of clusters, cluster number and Drugs acting on DTs.

Supplementary Table S3. The list of hyperbolically close DT pairs with their respective common interactors. Columns indicate protein identifier (gene symbol), metabolic pathways, Hyperbolic distance , and GO Term of DT pairs and their common interactors (DT_a, common interactor, DT_b, Hyperbolic distance between DT pairs, pathways of DT_a, pathways of common interactors, pathways of DT_b, GO Term of DT_a, GO Term of common interactors, GO Term of DT_b).
