# Supplementary figures and images for: Selection of Multi-Drug Targets against Drug-Resistant Mycobacterium tuberculosis XDR1219 Using the Hyperbolic Mapping of the Protein Interaction Network
Source: Int J Mol Sci. 2023 Sep 13;24(18):14050. doi: 10.3390/ijms241814050 (PMC10530867; doi:10.3390/ijms241814050)

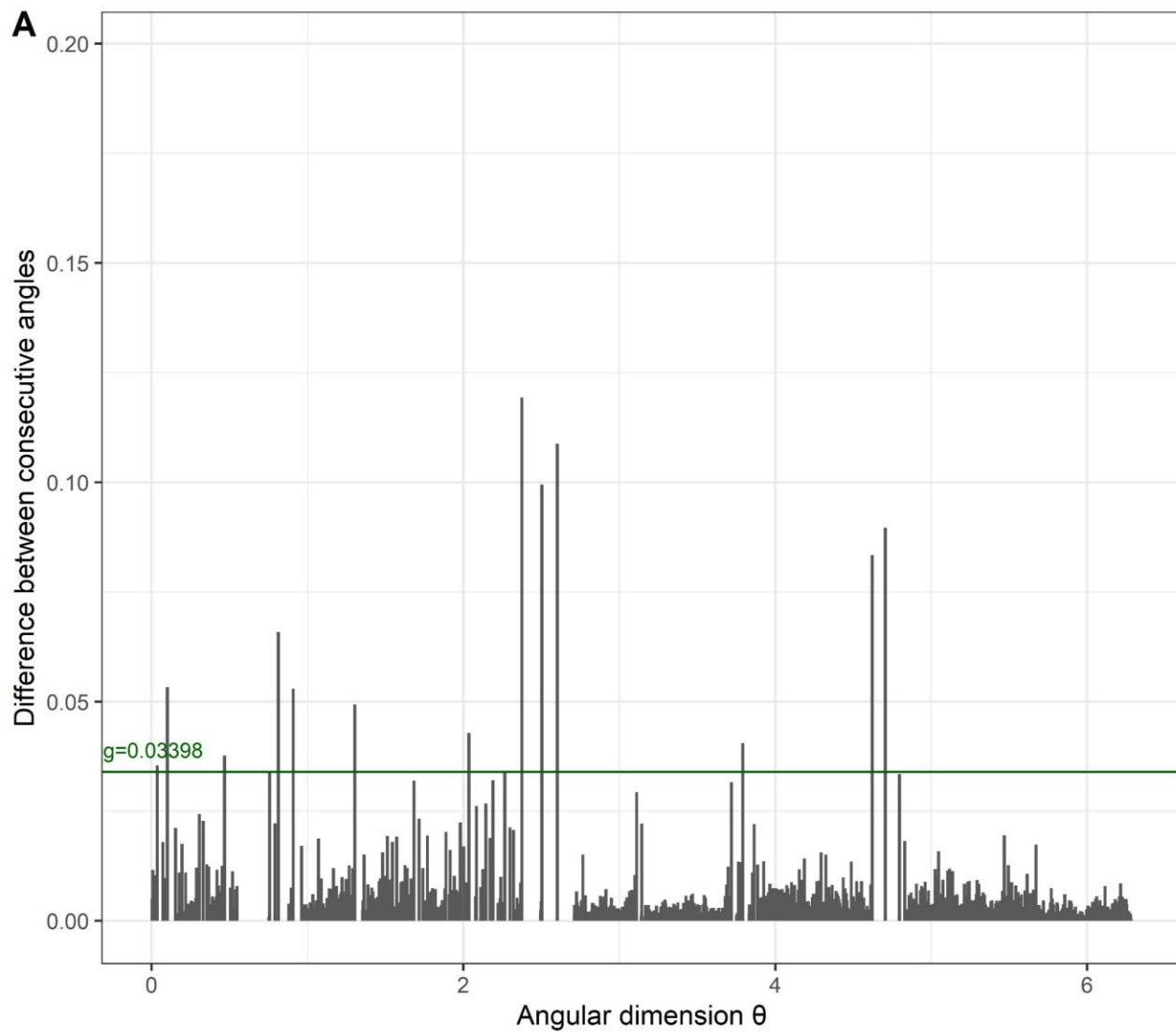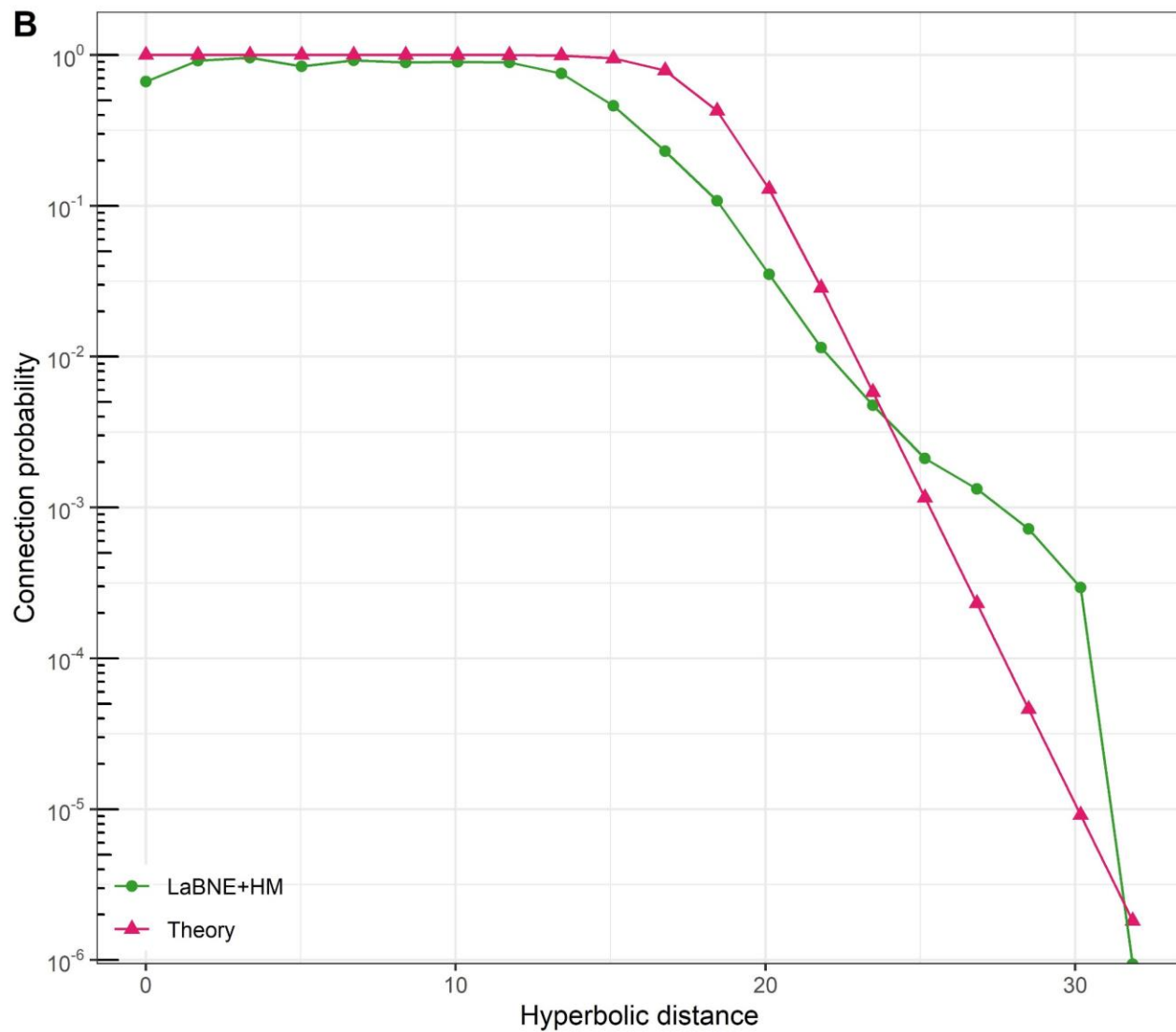

Supplement: Supplementary file 1 [file ijms-24-14050-s001.zip › SUPPLEMENTARY/Supplementary Figure S1.pdf]

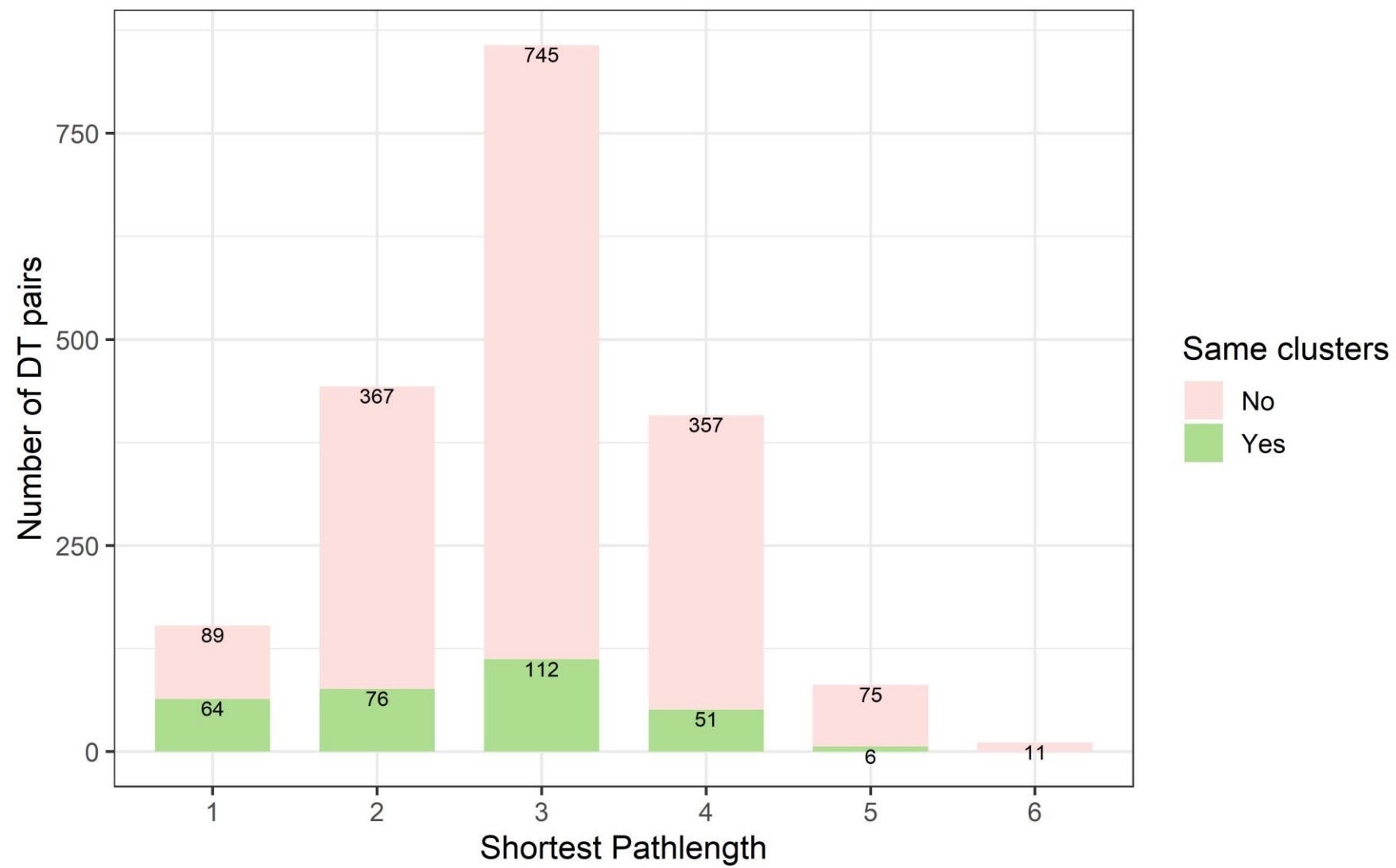

Supplement: Supplementary file 1 [file ijms-24-14050-s001.zip › SUPPLEMENTARY/Supplementary Figure S2.pdf]
